# The urine metabolome differs between lean and overweight Labrador Retriever dogs during a feed-challenge

**DOI:** 10.1371/journal.pone.0180086

**Published:** 2017-06-29

**Authors:** Josefin Söder, Ragnvi Hagman, Johan Dicksved, Sanna Lindåse, Kjell Malmlöf, Peter Agback, Ali Moazzami, Katja Höglund, Sara Wernersson

**Affiliations:** 1 Department of Anatomy, Physiology and Biochemistry, Swedish University of Agricultural Sciences, Uppsala, Sweden; 2 Department of Clinical Sciences, Swedish University of Agricultural Sciences, Uppsala, Sweden; 3 Department of Animal Nutrition and Management, Swedish University of Agricultural Sciences, Uppsala, Sweden; 4 Department of Molecular Sciences, Swedish University of Agricultural Sciences, Uppsala, Sweden; Faculty of Animal Sciences and Food Engineering, University of São Paulo, BRAZIL

## Abstract

Obesity in dogs is an increasing problem and better knowledge of the metabolism of overweight dogs is needed. Identification of molecular changes related to overweight may lead to new methods to improve obesity prevention and treatment. The aim of the study was firstly to investigate whether Nuclear Magnetic Resonance (NMR) based metabolomics could be used to differentiate postprandial from fasting urine in dogs, and secondly to investigate whether metabolite profiles differ between lean and overweight dogs in fasting and postprandial urine, respectively. Twenty-eight healthy intact male Labrador Retrievers were included, 12 of which were classified as lean (body condition score (BCS) 4–5 on a 9-point scale) and 16 as overweight (BCS 6–8). After overnight fasting, a voided morning urine sample was collected. Dogs were then fed a high-fat mixed meal and postprandial urine was collected after 3 hours. Metabolic profiles were generated using NMR and 45 metabolites identified from the spectral data were evaluated using multivariate data analysis. The results revealed that fasting and postprandial urine differed in relative metabolite concentration (partial least-squares discriminant analysis (PLS-DA) 1 comp: R^2^Y = 0.4, Q^2^Y = 0.32; cross-validated ANOVA: *P* = 0.00006). Univariate analyses of discriminant metabolites showed that taurine and citrate concentrations were elevated in postprandial urine, while allantoin concentration had decreased. Interestingly, lean and overweight dogs differed in terms of relative metabolite concentrations in postprandial urine (PLS-DA 1 comp: R^2^Y = 0.5, Q^2^Y = 0.36, cross-validated ANOVA: *P* = 0.005) but not in fasting urine. Overweight dogs had lower postprandial taurine and a trend of higher allantoin concentrations compared with lean dogs. These findings demonstrate that metabolomics can differentiate 3-hour postprandial urine from fasting urine in dogs, and that postprandial urine metabolites may be more useful than fasting metabolites for identification of metabolic alterations linked to overweight. The lowered urinary taurine concentration in overweight dogs could indicate alterations in lipid metabolism and merits further investigation.

## Introduction

Obesity is an increasing problem in the pet dog population [1, 2], mimicking the human situation worldwide. Overweight dogs suffer from obesity-associated conditions such as early onset of chronic diseases, decreased quality of life and a shortened life span [3, 4], strongly motivating research within the area. Overweight dogs have been shown to display metabolic alterations [5–7], many of which are also found in obese humans [8]. The origin of obesity is complex, with environmental and lifestyle factors with a polygenetic background being suggested to contribute [9]. An appetite-regulating genetic mutation has recently been discovered in Labrador Retriever dogs [10, 11], but the pathogenesis of dog obesity is far from understood.

Investigations of the metabolism of obese phenotypes using metabolomics have shown promising results in human and rodent models [12, 13]. A number of studies suggest that urine metabolites can discriminate between lean and overweight subjects [14, 15], revealing metabolites associated with obesity and potentially altered metabolic pathways. In dogs, voided urine samples can be collected non-invasively at home as well as in a hospital environment [16, 17] and urine is therefore both a practical and informative bio fluid for metabolic studies. Canine urine metabolite profiles have been successfully analysed, showing associations with breed, age and diet restriction [18–20]. However, to the best of our knowledge, no previous study has reported the use of urine metabolomics to analyse postprandial samples or alterations associated with overweight in dogs.

Most of the metabolomics data currently available represent the fasting state, although metabolism is a highly dynamic process. Research in humans has proven that the use of challenges, such as food intake or exercise, may increase inter-individual variations and reveal metabolite alterations not detectable in fasting conditions [21, 22]. Discrimination between lean and overweight dogs may thus be more feasible by studying the postprandial state in addition to the fasting state.

In this study, nuclear magnetic resonance (NMR) spectroscopy was combined with multivariate analysis in a feed-challenge for evaluation of relative urine metabolite concentrations in healthy Labrador Retriever dogs. The aim of the study was firstly to investigate whether NMR-based metabolomics could be used to differentiate postprandial from fasting urine in dogs, and secondly to investigate whether metabolite profiles differ between lean and overweight dogs in fasting and postprandial urine, respectively.

## Material and methods

### Animals

The study population consisted of 28 privately-owned intact male show-type Labrador Retriever dogs. To qualify for inclusion in the study, each dog had to be considered healthy by its owner and pass the health examination outlined below. Exclusion criteria consisted of historic or current systemic or organ-related diseases or treatment with antibiotics, non-steroid anti-inflammatory drugs, steroids, deworming drugs and proton pump inhibitors within three months prior to the examination day. This prospective observational study was performed at the Swedish University of Agricultural Sciences, Uppsala, Sweden. Dogs were recruited by personal letter to 715 owners (mostly located within 100 kilometres of Uppsala) of potentially eligible male Labrador Retrievers registered by The Swedish Kennel Club. Recruitment and data collection were performed during one year and all dogs were sampled once at the same time of the day according to a pre-designed protocol. Sixty owners replied and their dogs were examined for eligibility by an on-line survey of the dogs’ health status and their feeding and exercise routines. Thirty-two dogs were not invited for further data collection, based on the exclusion criteria. The remaining 28 dogs were invited to participate in the study and underwent data collection during the feed-challenge test.

### General study design

Dietary history was acquired and the frequency with which dogs were given table scraps and rewarded with training treats and dog chews were scored for each dog (Table 1). No adjustments were made to the dogs’ regular home diets of dry or wet complete dog food and treats prior to participation in the study. The dogs were fasted from 6 pm the day before the clinical samplings. In the morning of the examination day, water was withheld and a voided urine sample was taken from each dog by the owner before leaving home. On arrival at the clinic (between 8 and 9:30 am), the dogs were examined by the same veterinarian (JS) and fasting blood samples for health assessment were taken, followed by intake of a test meal. Postprandial urine samples were collected after 3 hours. The study was approved by the Ethical Committee for Animal Experiments, Uppsala, Sweden (C180/12), and the owner’s written consent was obtained for all dogs. The study followed guidelines for reporting observational studies in epidemiology [23].

**Table 1:** Dietary history of the 28 Labrador Retriever dogs included in the study^a^.

Dietary history	Lean dogs	Overweight dogs
	BCS (4–5)	BCS (6–8)
Frequency of scraps, treats and chews^b^	6 (4.5–7)	6 (3.5–7)
*pooled scores*, *median (interquartile range)*		
Daily energy intake from commercial diet^c^	2:10	2:14
*(n*_*75%*_:*n*_*100%*_*)*		
Commercial diet (*n*_wet_:*n*_dry_)^d^	1:11	0:16

^a^Summary of the background diet received by the dogs in their home environment. Body condition score (BCS) was evaluated by the same veterinarian (JS) and the dogs were divided into lean (n = 12) and overweight (n = 16) groups.

^b^The frequencies with which dogs were given table scraps and rewarded with training treats and dog chews were scored separately as follows: 0 (never),1 (once a month), 2 (1–3 times per week), 3 (daily). Scores for scraps, treats and chews were then pooled for each dog and the median (interquartile range) for the lean and overweight groups was calculated. The Mann-Whitney U test revealed no significant differences in total scores between BCS groups (*P* = 0.79).

^c^The proportion (%) of total daily energy intake coming from a commercial complete diet was estimated by the dog owner.

^d^Number of dogs fed wet or dry commercial diet in the home environment.

### Assessment of health status and body condition

Each dog underwent a physical examination, including general condition, skin condition, rectal temperature, mucus membranes, lymph nodes, heart and lung auscultation, abdominal palpation and gait. The dogs were weighed and photographed. Routine haematology (LaserCyte Hematology System, IDEXX Laboratories Inc, Westbrook, US) and serum biochemistry analyses (Architect c4000, Abbott Park, IL, US) of liver, kidney and thyroid functions, proteins, fructosamine, C-reactive protein and electrolytes (sodium, potassium and chloride) were performed on fasting blood samples. Urine was analysed by a standard dipstick chemistry test (Test strips for urine, Krulab, Langeskov, Denmark) and by refractometer for determination of urine specific gravity (Refractometer Master-URC/NOx, Atago, Bellevue, USA). Urine creatinine concentrations were analysed by an ELISA (Canine Urinary Creatinine ELISA, Arbor, Michigan, US). Dogs were assigned a clinical body condition score (BCS) according to a 9-point scale [24] (where fat deposits, waist and abdominal tuck are evaluated) by the same veterinarian (JS). The cut off for overweight as suggested by the scoring scale (BCS≥6) was applied. For BCS verifications, the fat cell hormone leptin was analysed in fasting serum (Canine Leptin ELISA, Millipore, Missouri, US) [25, 26]. Dogs with BCS 4–5 were considered lean and dogs with BCS 6–8 overweight. The lean group consisted of twelve dogs (mean ± SD, age 5.3±1.4 years, body weight/ideal body weight 34.8±2.5 kilograms) and the overweight group consisted of 16 dogs (age 5.2±1.6 years, body weight 39.5±4.6 kilograms, ideal calculated lean body weight 36.2±3.3 kilograms).

Based on BCS, 12 dogs were classified and grouped as lean (BCS 4–5) and 16 as overweight (BCS 6–8) [24]. As described previously [7], fasting serum leptin concentration was used to verify clinical body condition scoring [25, 26]. Mean ± standard deviation (SD) leptin concentration (ng/ml) was significantly higher (*P* = 0.048) in the overweight group (5.7±3.5) than in the lean group (3.4±1.9). It was confirmed that body weight was significantly different between the two groups of dogs (*P* = 0.004), while age and ideal lean body weight did not differ significantly.

### Urine sample collection and feed-challenge test

Urine was collected by the dog owners using a free-catch sampling device (Uripet, Rocket Medical, Washington, UK). Prior to the examination day, the dogs had experienced the urine sampling procedure at least three times, to accustom them to the procedure. On the examination day, naturally voided morning urine was collected at home and kept chilled on ice during transport. At the clinic, urine samples were centrifuged at 2000xg for 5 minutes at +4°C. The urine samples were then filtered (Filtropur S 0.2 um, Sarstedt AG & Co, Nümbrecht, Germany), transferred to polypropylene tubes (SC Micro Tube PCR-PT, Sarstedt AG & Co, Nümbrecht, Germany) and immediately frozen at -70°C. Three hours after the test meal, postprandial urine was collected at the clinic and directly treated and frozen in the same manner as above.

All dogs were exposed to the same feed-challenge test, in which they were given half their daily energy requirement as a high-fat mixed-meal. Daily energy requirement was calculated based on actual body weight (BW) in lean dogs and on calculated ideal lean body weight [24, 27] in overweight dogs. The equation used to compute daily energy requirements (131 kcal x BW_kg_^0.75^) is designed specifically for adult intact Labrador Retrievers [28]. The lean dogs received 222±12 g of the test meal and the overweight dogs received 228±16 g (mean ± SD, no significant difference between groups *P* = 0.22). The test meal was weighed and served with water added to the meal (same amount in grams as the individual test meals). The test feed (Science Plan^TM^ Canine Adult Performance, Hills, Etten Leur, the Netherlands) provided 4230 kcal/kg, with 51% of the metabolisable energy (ME) as fat, 26% as carbohydrate and 23% as protein (taurine, omega-3 and omega-6 fatty acids, betacarotene and vitamin A, D, E and C were added by the manufacturer). Nutrient composition and energy content of the test feed were confirmed by an independent authorised laboratory (Food & Agro Testing Sweden AB, Eurofins, Lidköping, Sweden). The postprandial period started at the first bite and all 28 dogs voluntarily consumed all food and water within 10 minutes of being served. The dogs were given nothing further to eat or drink and were kept indoors until 3 hours after the test meal. They were then taken outside and a naturally voided postprandial urine sample was collected.

### NMR spectral acquisition and identification of urine metabolites

Prior to NMR spectral acquisition, the frozen urine samples were thawed at 6°C and from each sample 150 μl of urine were withdrawn and mixed with 150 μl phosphate buffer (150 mM, pH 7.4, 0.01% TSP (3-(trimethylsilyl)-2,2',3,3'-tetradeuteropropionic acid)) in a 3 mm NMR tube. The NMR analyses were performed on a Bruker Avance II 600MHz spectrometer equipped with a QCI H-C/P/N-D cryoprobe and a SampleJet sample changer with a sample cooling system (Bruker Biospin AG, Fällanden, Switzerland).

For spectral acquisition and processing the software TopSpin 3.1, supplied by Bruker Biospin AG, was used. A one dimensional noesy sequence with water presaturation was used for acquisition, with a relaxation delay of 4 seconds and 128 scans for each experiment. All measurements were made at 25°C. After acquisition, each spectrum was Fourier-transformed following multiplication by line broadening of 0.5 Hz, phase corrected, baseline corrected and referenced to TSP at 0.0 ppm.

The spectra were then analysed using the ChenomX NMR suite 7.5 database (Chenomx Inc., Edmonton, Canada). A total of 47 metabolites were identified in each spectrum using the ChenomX database and their concentrations were quantified in mM relative to the internal standard added (TSP), after accounting for overlapping signals. As creatinine and urea were present at much higher concentrations than other metabolites (creatinine up to 1000-fold higher and urea up to 5000-fold higher), any small change in their concentrations would have risked causing a large change in the relative ratio of other metabolites. These two metabolites were therefore excluded from the datasets, after which 45 metabolites remained. To account for different concentrations in the urine samples, the data from both fasting and postprandial time points were transformed to relative concentrations (% of total mM) by using the following formula: [(mM of metabolite)/(sum of mM of all 45 metabolites)] x100. All metabolite concentrations were therefore relative and are referred to hereafter as “relative concentrations”.

### Statistical analyses

**Fig 1:**
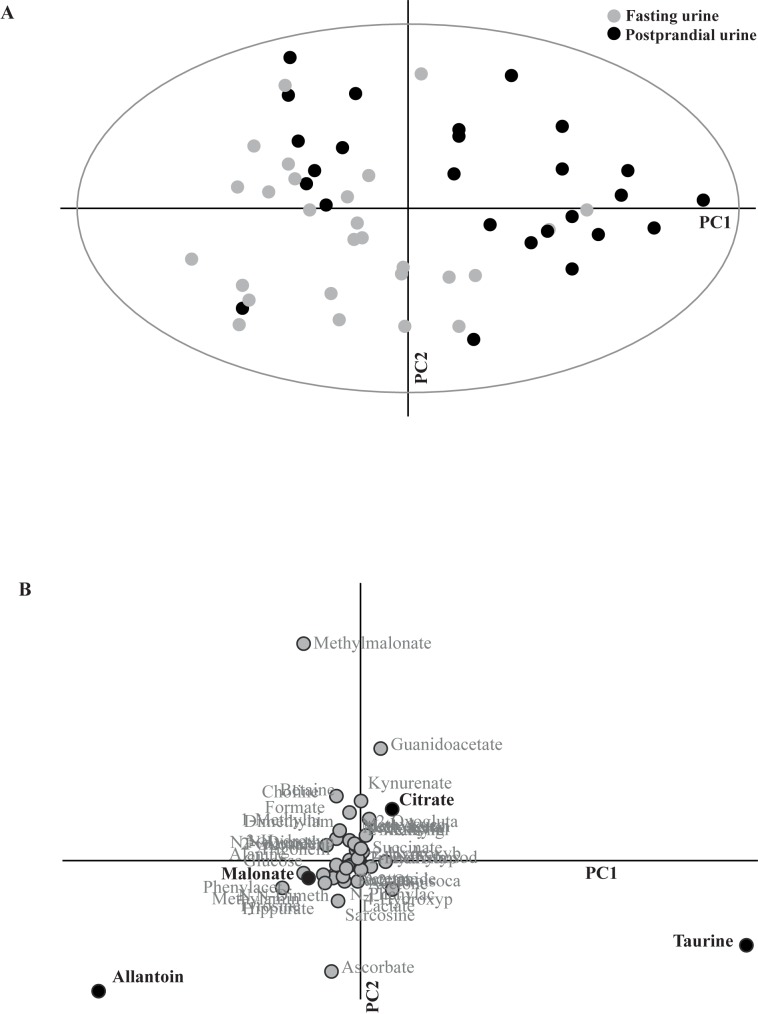
Principal component analysis between fasting and postprandial time points. Fasting and postprandial urine samples showed a clear separation in principal component analysis (A). All 45 metabolites and all 28 dogs were included in this unconstrained model. Principal component (PC) 1 explained 12% of the variance and PC2 7%. The separation proved significant in a partial least-squares discriminant analysis (PLS-DA) model (for *P*-values, see Results section) where fasting and postprandial urine samplings were pre-defined as two separate groups. Loading plot (B) corresponds to the principal component analysis (A). Discriminative metabolites making a significant contribution to the separation between fasting and postprandial urine in the PLS-DA model (allantoin, taurine, citrate and malonate) are highlighted in bold text and with black dots.

**Fig 2:**
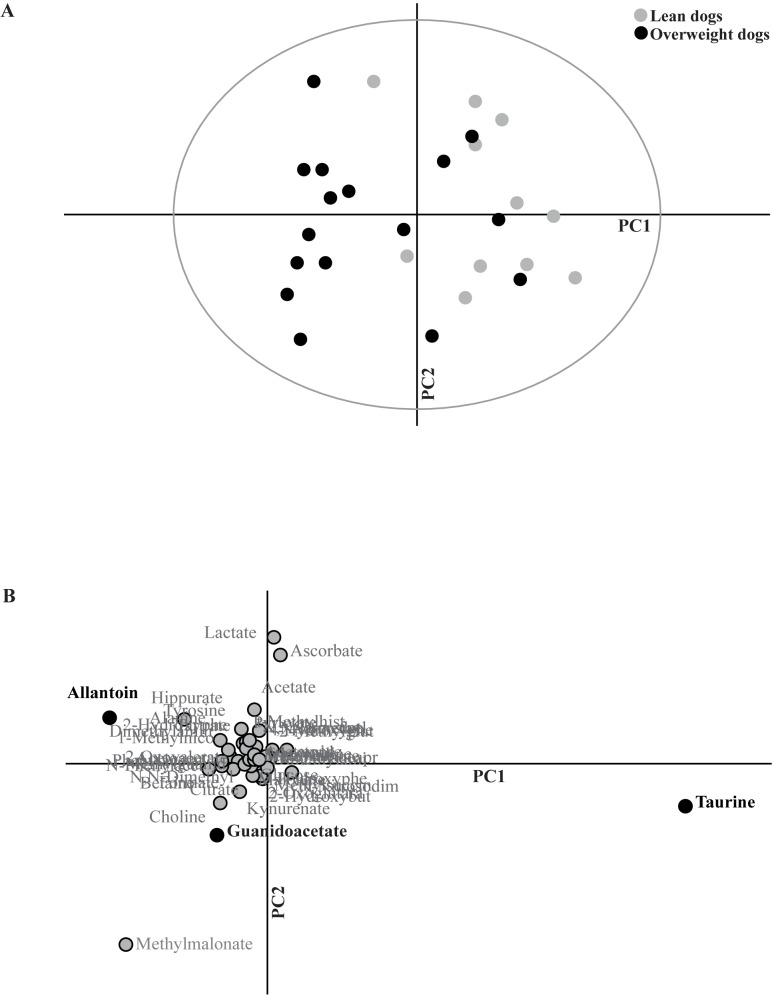
Principal component analysis between lean and overweight Labrador Retriever dogs in postprandial urine. Lean and overweight dogs showed a clear separation in principal component analysis of the postprandial urine dataset (A). All 45 metabolites were included in this unconstrained model. Principal component (PC) 1 explained 8% of the variance and PC2 4%. The separation proved significant in a partial least-squares discriminant analysis (PLS-DA) model (for *P*-values, see Results section) where lean (n = 12) and overweight dogs (n = 16) were pre-defined as two separate groups. Loading plot (B) corresponds to the principal component analysis (A). Discriminant metabolites making a significant contribution to the separation between lean and overweight dogs in the PLS-DA model (taurine, allantoin and guanidoacetate) are highlighted in bold text and with black dots.

Paired and unpaired t-tests, the Wilcoxon signed-rank test and the Mann-Whitney U test were used to compare normally and non-normally distributed data (age, body weight, ideal lean body weight, fasting leptin concentrations, urine creatinine concentrations, dietary history and urine specific gravity) between groups or time points (GraphPad Prism 5.0, San Diego, California, US). A value of *P*<0.05 was considered significant in statistical analyses unless otherwise indicated. In the multivariate analysis presented, the ellipse in Figs 1 and 2 was set at 95% Confidence interval (CI).

Multivariate comparisons were performed on datasets containing relative urine metabolite concentrations (n = 45 as x-variable) using commercially available software (SIMCA-P + 13.0 Umetrics, Umeå, Sweden). Randomisation of raw data, pareto-scaling and step-wise removal of up to three outliers in each comparison was applied. Principal component analysis (PCA) was used to visualise any unconstrained clustering of fasting and postprandial time points (including all 28 dogs), as well as any clustering of lean and overweight dogs within each time point. In an unconstrained multivariate model, as in a PCA, the computer program is unaware of any pre-designed grouping of the samples, which enables an unconditional and exploratory approach to the data. The observed clustering was thereafter tested for significance with a constrained partial least-squares discriminant analysis (PLS-DA). In a constrained multivariate model, the computer program is aware of the grouping and/or clustering of the samples and can answer if there are any differences between treatments. The PLS-DA model was further investigated using cross-validated analysis of variance (CV-ANOVA) [29] for further verification of the multivariate model. Variable importance for the projection (VIP) based on the PLS-DA was used to determine the most important discriminative metabolites in each comparison. Discriminative metabolites identified using the PLS-DA models were then visualised in the loading plots corresponding to each PCA. Metabolites with VIP *>*1 and for which the corresponding jackknife-based 95% CI were not close to or including zero were considered discriminative and significant for the observed separations.

Discriminative metabolites identified by the multivariate approach were further investigated by univariate data analysis (GraphPad Prism 5.0, San Diego, California). This analysis was performed to enable a physiological interpretation of the results, i.e. to determine whether the urine concentrations of the discriminant metabolites were increased or decreased between time points or, whether they were higher or lower in lean or overweight dogs, respectively. The Wilcoxon signed-rank test and Mann-Whitney U test were used for non-normally distributed paired and unpaired comparisons of relative metabolite concentrations between fasting and postprandial time points (including all 28 dogs) and between lean and overweight dogs at the postprandial time point. Notably, only the discriminative metabolites identified in PLS-DA models were included in the univariate statistical analyses and Bonferroni corrections (*P* = 0.05/number of discriminative metabolites or comparisons) were performed to counteract problems with multiple comparisons.

## Results

### Health status

For the 28 essentially healthy Labrador Retrievers included in the study, no clinically noteworthy abnormalities were detected by haematology or serum biochemistry. Urine standard dipstick chemistry was within the reference range for healthy dogs, as was urine specific gravity (mean ± SD) which was 1.034±0.01 in fasting samples and 1.032±0.01 in postprandial samples. Urine specific gravity was not significantly different between time points or between lean (1.036±0.01) and overweight dogs (1.033±0.01) in fasting urine. Mean ± SD creatinine concentrations (mM) measured by ELISA were not significantly different in fasting (17.2±7.0) compared to postprandial urine (18.4±7.8). Vital parameters at physical examination were within the reference range for healthy dogs, but minor health problems were found in 11 dogs, e.g. slightly stiff gait and mild lameness, signs of periodontitis, palpable peri-articular osteophyte formation and skin furunculosis. None of the dogs exhibiting these minor health problems was excluded, as vital parameters were normal. There were no missing values in any of the data collected on the 28 included dogs. No significant differences were found in dietary history between lean and overweight dogs (Table 1).

### Comparison of fasting and postprandial time points

There was a clear separation between fasting and postprandial time points in relative metabolite concentrations in the multivariate PCA model of all 28 dogs (Fig 1A). Significant separation was also confirmed in the PLS-DA model using the two time points as pre-defined groups (PLS-DA 1 comp: R^2^Y = 0.4, Q^2^Y = 0.32; CV-ANOVA: *P* = 0.00006). Using VIP analyses, discriminant metabolites between fasting and postprandial time points were identified as allantoin, taurine, citrate and malonate (Table 2). Comparing the PCA plot (Fig 1A) with the corresponding loading plot (Fig 1B) revealed that taurine and citrate concentrations had increased and allantoin and malonate concentrations had decreased in postprandial compared with fasting urine. The univariate analyses were consistent with the multivariate models with the exception of malonate that was not significant after Bonferroni correction (*P*<0.013), although there was a trend of decreased malonate concentrations (*P* = 0.044) (Table 2). The mean taurine (mM)/creatinine (mM) ratio for all dogs at the fasting time point was 0.099 (95% CI: 0.06, 0.14).

**Table 2:** Discriminant metabolites between fasting and postprandial time points in the 28 included Labrador Retriever dogs.

Metabolite	Relative concentration^a^		VIP (CI)^b^	*P*-value^c^
	Fasting urine	Postprandial urine		
Taurine^d^	11.2 ± 9.21	15.2 ± 10.7	2.9 (1.7)	0.0005
Allantoin	24.0 ± 5.71	16.2 ± 6.80	4.7 (0.9)	<0.0001
Citrate	1.06 ± 0.61	2.59 ± 2.42	1.7 (1.3)	<0.0001
Malonate	3.10 ± 1.65	2.36 ± 1.72	1.2 (0.8)	0.044

^a^Relative concentrations were calculated by normalisation of the molar concentration of each metabolite to the total molar concentration of all 45 metabolites for each dog (values presented as *Mean ± SD (% of total mM))*.

^b^VIP, Variable importance for the projection; CI, confidence interval.

^c^The Wilcoxon signed-rank test was used for univariate analyses of identified discriminant metabolites between time points. Level of significance *P*<0.013 after Bonferroni correction.

^d^Relative taurine urinary concentrations increased by 36% from fasting to the postprandial time point.

### Comparison of lean and overweight dogs

**Fig 3:**
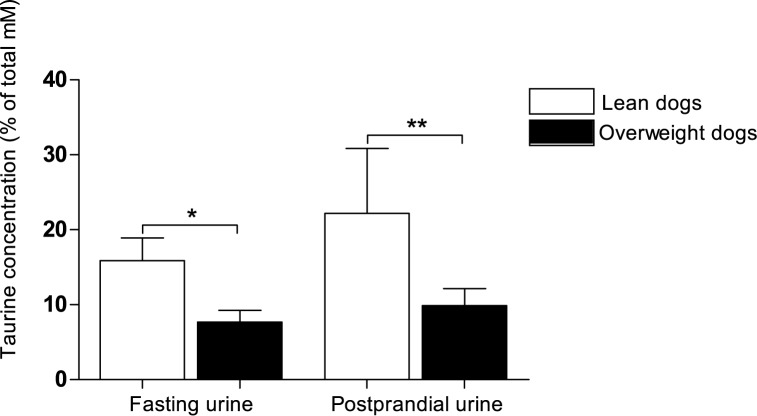
Relative taurine concentrations in fasting and postprandial urine in lean and overweight Labrador Retriever dogs. Body condition score (BCS) was evaluated by the same veterinarian (JS) and 12 lean (BCS 4–5) and 16 overweight (BCS 6–8) Labrador Retriever dogs were subjected to a feed-challenge. Relative taurine concentrations were calculated by normalisation of the molar concentration of taurine to the total molar concentration of all 45 metabolites in each dog´s sample (values presented as *Mean ± SD (% of total mM))*. The Mann-Whitney U test was used for comparisons between lean and overweight groups of dogs at the fasting and postprandial time point, respectively. Level of significance *P*<0.025 after Bonferroni correction. Significant differences between groups are marked with asterisks (* *P* = 0.022) and (** *P* = 0.006).

At the fasting time point, no separation was observed between body condition groups in the multivariate PCA model. This lack of difference was confirmed both by a PLS-DA model (1 comp: R^2^Y = 0.38, Q^2^Y = 0.01) and by a CV-ANOVA (*P* = 0.87). At the postprandial time point, however, there was a separation in relative metabolite concentrations between lean and overweight dogs in the PCA model (Fig 2A). This postprandial separation between lean and overweight dogs was verified in a PLS-DA model using lean and overweight dogs as pre-defined groups (PLS-DA 1 comp: R^2^Y = 0.5, Q^2^Y = 0.36; CV-ANOVA: *P* = 0.005). Using VIP analyses, discriminant postprandial metabolites between lean and overweight dogs were identified as taurine, allantoin and guanidoacetate (Table 3). Comparing the PCA plot (Fig 2A) with the corresponding loading plot (Fig 2B) showed that taurine concentration was lower, whereas allantoin and guanidoacetate concentrations were higher in overweight than in lean dogs. Univariate analyses demonstrated that taurine concentration was significantly lower in overweight than in lean dogs. Moreover, there was a trend of higher allantoin concentration in overweight dogs (*P* = 0.031), although this difference was not significant after Bonferroni correction (*P*<0.017). Guanidoacetate concentration was not significantly different postprandially between body condition groups in the univariate comparison (Table 3). Relative taurine concentrations in fasting and postprandial urine in lean and overweight dogs are displayed in Fig 3.

**Table 3:** Discriminant metabolites between lean and overweight Labrador Retriever dogs in postprandial urine.

Metabolite	Relative concentration^a^		VIP (CI)^b^	*P*-value^c^
	Lean dogs	Overweight dogs		
	BCS (4–5)	BCS (6–8)		
Taurine	22.2 ± 8.65	9.89 ± 9.07	5.0 (1.2)	0.006
Allantoin	12.8 ± 7.16	18.7 ± 5.47	2.4 (1.0)	0.031
Guanidoacetate	5.73 ± 2.25	7.49 ± 2.41	1.4 (0.9)	0.11

Body condition score (BCS) was evaluated by the same veterinarian (JS) and the dogs were divided into lean (n = 12) and overweight (n = 16) groups.

^a^Relative concentrations were calculated by normalisation of the molar concentration of each metabolite to the total molar concentration of all 45 metabolites for each dog (values presented as *Mean ± SD (% of total mM))*.

^b^VIP, Variable importance for the projection; CI, confidence interval.

^c^The Mann-Whitney U test was used for univariate analyses of identified discriminant metabolites between lean and overweight groups. Level of significance *P*<0.017 after Bonferroni correction.

## Discussion

In this study, we used NMR-based metabolomics to analyse urine metabolite profiles in healthy male Labrador Retriever dogs during a feed-challenge test. We found a clear separation between fasting and postprandial urine samples, demonstrating that this methodology can be used for urine analyses when investigating metabolic events in response to food intake in dogs. Moreover, using multivariate modelling significant differences in metabolite profiles between lean and overweight dogs were seen in postprandial, but not in fasting urine. These results suggest that metabolic alterations related to overweight and obesity in dogs may be more prominent in postprandial than in fasting events, which is in agreement with general findings from human studies [21, 22]. For example, Krug *et al* demonstrated that discrete metabotypes not observed in the normal fasting state could be revealed after various metabolic challenges in healthy volunteers [22] and Pellis *et al* were able to identify obesity related metabolic changes in postprandial plasma that were not detectable in fasted non-perturbed conditions [21].

The increased relative concentration of taurine found in postprandial urine compared to fasting confirms earlier findings in dogs [30]. Taurine is a sulphur-amino acid involved in a variety of body functions, including fat metabolism, reproduction and the nervous system. Taurine is freely filtered in the kidneys [31]. A previous study could not find any compensatory kidney reabsorption during prolonged fasting in dogs, although plasma concentrations slightly increased [30]. This is supporting the claim that plasma taurine concentration is crudely reflected in urine [31] but might be conserved in plasma during fasting. Taurine has an essential role in lipid metabolism as a bile acid conjugator, aiding fat absorption in the small intestine [32]. Increased intestinal release of bile taurine in response to the high-fat meal used in the present study was therefore expected with a subsequent increase also seen in urine. Dogs absorb taurine from their diet, but also have the capacity for taurine biosynthesis from cysteine and methionine in the liver [33]. It should be noted that in the present case taurine was added by the manufacturer to the test diet, and hence the increased taurine measured in postprandial urine could have originated both from the test meal itself and from endogenously synthesised taurine released in response to the meal. In the present study, the size of the test meal was not significantly different between lean and overweight dogs.

A major finding in this study was the significantly lower postprandial urinary taurine found in overweight compared with lean dogs. Univariate statistical tests identified a difference in relative taurine concentration between body condition groups also in fasting urine, although the multivariate PLS-DA model including all metabolites did not. In veterinary medicine, taurine deficiency has been associated with dilated cardiomyopathy in dogs of certain breeds [34]. Our findings suggest that reduced taurine concentrations could be linked also to increased adiposity in dogs, alternatively that lean dogs require less taurine and subsequently excrete more. A connection between taurine deficiency and overweight as suggested by our results, is well in line with findings in a number of studies in humans and rodents [35, 36]. In humans, urinary taurine excretion has been negatively associated with body mass index [37] and in rodent models of obesity, a lower concentration of urinary taurine has been found in obese individuals [38–40]. Moreover, it has been proposed that obesity induced by a high-fat diet can result in taurine depletion [36]. Compared to the findings by Gray *et al*, we found a lower relative increase in urinary taurine from fasting to postprandial time point [30]. These discrepancies between studies could be coupled to differences in fat content in the selected test diets. The higher fat content in the test diet used in the present study could have resulted in greater loss of gastrointestinal taurine in faecal bile. Although the composition of the test diet may have influenced the postprandial urinary excretion in the present study, lean and overweight dogs differed also in fasting taurine urinary excretion (as shown in Fig 3). The reason behind the reduced concentration of taurine in overweight dogs in the present study is unknown, and may involve both genetic and lifestyle factors. We cannot exclude the possibility of a history of a high-fat diet in the overweight group, which could possibly account for the decreased taurine concentration over time in these dogs. However, we were unable to identify any significant differences in feeding regime between the two groups of dogs based on statements from their owners. It has been suggested that taurine deficiency could have a negative effect on lipid metabolism, and thereby promote obesity and obesity-related disorders in a vicious cycle [36, 37]. Evidence in support of this suggestion is that dietary supplementation of taurine decreases body weight and serum lipid concentrations in rodents [36, 41] and humans [42], possibly by increasing fatty acid oxidation [36]. The lower taurine levels in overweight dogs could be connected to less efficient lipid turnover than in lean dogs. However, further studies are needed to clarify the role of taurine in dog obesity and to evaluate whether taurine supplementation could be beneficial for weight loss in obese dogs.

The decreased concentration of allantoin in postprandial compared with fasting urine suggests that body protein catabolism overnight exceeded dietary protein breakdown at three hours after food intake. Allantoin is formed by oxidation of uric acid and is produced as a result of purine metabolism in most mammals, including dogs, but not humans [43]. It should be noted that the morning urine samples in the present study contained the allantoin excreted during one night of withheld food, whereas three hours of post-absorptive state had elapsed at the postprandial samplings. It is possible that the allantoin concentration detected postprandially would have differed if the test feed had been a high-protein diet or if a sample from a later post-absorptive state had been tested. In the multivariate postprandial model, allantoin and guanidoacetate concentrations contributed to the separation of lean and overweight dogs. Guanidoacetate is present in the metabolism of amino groups of many amino acids and is therefore also involved in protein metabolism [44]. The trend of higher postprandial concentrations of these two metabolites in overweight than in lean dogs could be associated with a metabolism directed towards amino acids instead of lipids. Alternatively, the catabolism of amino acids and proteins might be upregulated in this overweight group of dogs. These findings should be interpreted cautiously, but in combination with possible taurine depletion, this could indicate that the overweight dogs are slower than lean dogs in shifting from protein catabolism at fasting to lipid metabolism after consumption of a high-fat meal.

The increased relative concentration of postprandial urinary citrate observed in the present study could be interpreted as normal energy metabolism. The tricarboxylic acid cycle, in which citrate is created, generates energy from carbohydrates, proteins and fats, components all supplied by the test meal. It has been suggested that citrate could accumulate in plasma the first hours after a meal intake due to the ATP converting capacity of the tricarboxylic acid cycle [21]. In kidneys, citrate is suggested to be freely filtered and thereafter almost completely reabsorbed in canine tubules [45]. Higher energy status in dogs after the meal could explain the increased concentrations of citrate found in postprandial urine, possibly caused by incomplete renal reabsorption following increased postprandial plasma citrate concentrations. Citrate concentrations are also regulated by hepatic clearance and influenced by insulin, glucose and free fatty acid concentrations [46]. Interestingly, it has been shown in rodent models that obesity and insulin resistance can induce either increased or decreased urinary citrate concentrations [14, 47]. In the present study we did not find any association between body condition and relative urinary citrate concentration in fasting or postprandial samples. This could be explained by the fact that none of the dogs, as earlier described [7], exhibited profound insulin resistance and that they were overweight rather than obese. The trend for decreased concentration of malonate in postprandial compared with fasting urine could, as citrate, reflect a modulation of body energy homeostasis. Urinary malonate is thought to originate from hydrolysed tissue malonyl-CoA [48], a metabolite responsible for the interplay between fatty acid synthesis and oxidation [49]. Subsequently, malonyl-CoA is considered a key regulator in fatty acid metabolism. It is therefore likely that the altered urinary malonate concentration is a consequence of the high-fat meal consumed in the feed-challenge.

Normalisation of urine data is crucial for comparisons of metabolite concentrations between different time points or studies. Traditionally, normalisation to creatinine has been used to account for differences in urine concentrations [50, 51]. However, creatinine concentration in dog urine is not only affected by variations in urine density but may also be influenced by other factors, e.g. body weight and food intake [52]. This could have a negative impact considering the layout of the present study. Moreover, the suitability of creatinine as a general normalisation factor in urine metabolomics has been questioned [53] especially in analyses of postprandial urine as it was found that meat-based food intake increases the creatinine excretion. An alternative approach for accounting for differences in urine volume is to integrate each NMR spectrum into defined (e.g. 0.01-ppm) integral regions and then normalise each spectral region to the total spectral intensity. However, such an approach cannot account for overlapping signals of different metabolites within each spectral region. In the present study, these overlaps were accounted for before normalisation by calculating the concentration (mM) of each metabolite in the urine samples using ChenomX profiler in spectral data. The concentration of each metabolite was thereafter calculated relative to the total molar concentration of all 45 metabolites for each dog, in order to account for differences in urine concentrations. This approach for normalisation [40] commonly used in metabolomics studies, is comparable to using creatinine ratios, as was also demonstrated in a life-long dog study including spot urine samples [19]. We are aware that relative metabolite concentrations cannot easily be used for comparisons with studies using other methods for normalisation. But, in the present case the normalisation approach enabled comparison between fasting and postprandial sampling time points, while at the same time accounting for overlapping signals and differences in urine volume.

### Study limitations

In previous metabolomics studies on urine from dogs, certain factors that may contribute to variance in the methodology, such as breed, age and feeding regime have been identified [18–20]. Based on this information, the design of the present study focused on eliminating these factors by using clinically healthy dogs of the same breed with a narrow age range and exposing them to a standardized feed challenge. It should be noted that total emptying of the bladder after morning urination could not be confirmed, as the dogs were still at home at that time. All urine samples were collected by natural voiding and it is possible that there could have been a mix of fasting and postprandial urine in the postprandial sample obtained from some dogs. This is a limitation of the study, however, the multivariate model was able to find clear differences between the fasting and 3-hour postprandial time points. In humans, the 2–4 hour postprandial time frame has been identified as a stable window for comparison with fasting samples [53], and it is possible that this could also apply for canine metabolism as well as suggested by our results. If incomplete emptying of the bladder has occurred at the morning urination we see no reason why this should have differed between lean and overweight groups of dogs and should in that way not have influenced the results negatively. After thorough consideration we decided to include dogs with BCS 6 in the overweight group, although clinically these are considered only slightly overweight. The reasons for this decision include that these dogs constitute the majority of the dog population. Moreover, severely obese but healthy dogs (BCS 8–9) showed extremely difficult to enroll partly because these dogs also had other health problems. Despite the moderate differences in body condition status between the lean and overweight groups of dogs, significant differences were seen between groups in multivariate models of postprandial metabolite relative concentrations as well as in taurine relative concentrations in fasting and postprandial urine.

## Conclusions

This study demonstrated that NMR-based metabolomics can differentiate 3-hour postprandial urine from fasting urine and can thus be used for evaluation of metabolic events in response to food intake in dogs. After a feed challenge, we found differences in urine metabolite profiles between lean and overweight dogs coupled both to lipid and protein metabolism. Most of these differences were not detectable in fasting conditions, suggesting that postprandial urine metabolites may be more useful than fasting metabolites for identification of metabolic alterations linked to overweight and obesity in dogs. The lowered relative urinary taurine concentration detected in overweight dogs could indicate alterations in lipid metabolism thus, taurine in overweight dogs merits further investigation.

## Supporting information

S1 Table Relative concentrations of urine metabolites in fasting and postprandial samples from all dogs. (DOCX)

S2 Table Relative concentrations of urine metabolites in lean and overweight dogs at fasting and postprandial time points. (DOCX)
