# Supplementary material for: The urine metabolome differs between lean and overweight Labrador Retriever dogs during a feed-challenge
Source: PLoS One. 2017 Jun 29;12(6):e0180086. doi: 10.1371/journal.pone.0180086 (PMC5491113; doi:10.1371/journal.pone.0180086)
Supplement: S1 Table — (DOCX) [file pone.0180086.s001.docx]

**Supplementary Table 1. Relative concentrations of urine metabolites in fasting and postprandial samples from all dogs**

|  | **Relative concentration^a^**  *Mean ± SD (% of total mM)* | |  |  |
| --- | --- | --- | --- | --- |
| **Metabolite** | **Fasting (n=28)** | **Postprandial (n=28)** | **VIP (CI)^b^** | ***P*-value^c^** |
| 1-Methylnicotinamide | 1.74 ± 0.78 | 1.86 ± 0.65 | 0.2 (0.6) |  |
| 2-Hydroxybutyrate | 0.94 ± 0.22 | 1.11 ± 0.33 | 0.5 (0.3) |  |
| 2-Hydroxyphenylacetate | 0.54 ± 0.13 | 0.73 ± 0.31 | 0.5 (0.5) |  |
| 2-Methylglutarate | 0.33 ± 0.27 | 0.42 ± 0.31 | 0.1 (0.3) |  |
| 2-Oxoglutarate | 2.09 ± 0.79 | 2.30 ± 0.83 | 0.4 (0.9) |  |
| 2-Oxoisocaproate | 0.43 ± 0.08 | 0.50 ± 0.14 | 0.3 (0.2) |  |
| 2-Oxovalerate | 1.11 ± 0.30 | 1.04 ± 0.37 | 0.2 (0.3) |  |
| 3-Hydroxyphenylacetate | 0.39 ± 0.15 | 0.37 ± 0.14 | 0.05 (0.2) |  |
| 4-Hydroxyphenylacetate | 0.49 ± 0.29 | 0.61 ± 0.35 | 0.3 (0.5) |  |
| Acetamide | 0.62 ± 0.16 | 0.69 ± 0.18 | 0.3 (0.2) |  |
| Acetate | 1.38 ± 0.28 | 1.46 ± 0.61 | 0.2 (0.7) |  |
| Acetoacetate | 0.51 ± 0.07 | 0.61 ± 0.14 | 0.4 (0.2) |  |
| Acetone | 0.36 ± 0.11 | 0.37 ± 0.12 | 0.07 (0.2) |  |
| Alanine | 2.34 ± 0.67 | 1.85 ± 0.64 | 1.0 (0.1) |  |
| Allantoin | 24.0 ± 5.71 | 16.2 ± 6.80 | 4.7 (0.9) | <0.0001 |
| Ascorbate | 6.18 ± 2.74 | 4.70 ± 2.89 | 1.3 (1.7) |  |
| Betaine | 3.13 ± 1.67 | 3.23 ± 1.11 | 0.03 (0.8) |  |
| Choline | 1.37 ± 0.92 | 1.45 ± 0.94 | 0.1 (0.3) |  |
| Citrate | 1.06 ± 0.61 | 2.59 ± 2.42 | 1.7 (1.3) | <0.0001 |
| Dimethylamine | 1.44 ± 0.56 | 1.65 ± 0.45 | 0.4 (0.5) |  |
| Formate | 0.99 ± 0.60 | 1.05 ± 0.68 | 0.02 (0.3) |  |
| Glucose | 2.55 ± 1.17 | 2.14 ± 0.69 | 0.7 (0.2) |  |
| Guanidoacetate | 5.69 ± 2.33 | 6.73 ± 2.46 | 1.0 (1.0) |  |
| Hippurate | 2.08 ± 2.00 | 2.10 ± 1.61 | 0.04 (0.6) |  |
| Kynurenate | 2.70 ± 1.51 | 2.95 ± 1.76 | 0.06 (0.4) |  |
| Lactate | 3.07 ± 2.08 | 3.77 ± 3.44 | 0.7 (0.8) |  |
| Malonate | 3.10 ± 1.65 | 2.36 ± 1.72 | 1.2 (0.8) | 0.044 |
| Methylamine | 0.52 ± 0.20 | 0.35 ± 0.11 | 0.6 (0.3) |  |
| Methylguanidine | 0.73 ± 0.19 | 0.91 ± 0.23 | 0.5 (0.3) |  |
| Methylmalonate | 4.79 ± 3.55 | 6.57 ± 4.95 | 0.9 (1.5) |  |
| Methylsuccinate | 0.47 ± 0.42 | 0.65 ± 0.77 | 0.08 (0.5) |  |
| N,N-Dimethylformamide | 0.61 ± 0.14 | 0.57 ± 0.13 | 0.2 (0.2) |  |
| N,N-Dimethylglycine | 0.50 ± 0.30 | 0.37 ± 0.24 | 0.4 (0.2) |  |
| N-Nitrosodimethylamine | 2.26 ± 0.69 | 2.66 ± 0.96 | 0.7 (0.9) |  |
| N-Phenylacetylglycine | 2.34 ± 1.39 | 2.60 ± 1.56 | 0.2 (0.9) |  |
| Phenylacetate | 1.16 ± 0.34 | 1.14 ± 0.32 | 0.1 (0.3) |  |
| Pyruvate | 1.01 ± 1.96 | 0.61 ± 0.17 | 0.6 (1.0) |  |
| Sarcosine | 0.74 ± 0.66 | 0.40 ± 0.28 | 0.8 (0.4) |  |
| Succinate | 0.34 ± 0.13 | 0.39 ± 0.14 | 0.2 (0.4) |  |
| Tartrate | 0.69 ± 0.19 | 0.58 ± 0.18 | 0.4 (0.4) |  |
| Taurine | 11.2 ± 9.21 | 15.2 ± 10.7 | 2.9 (1.7) | 0.0005 |
| Trigonelline | 0.35 ± 0.22 | 0.28 ± 0.13 | 0.3 (0.4) |  |
| Trimethylamine | 0.17 ± 0.04 | 0.20 ± 0.05 | 0.2 (0.2) |  |
| Tyrosine | 0.82 ± 0.39 | 0.74 ± 0.38 | 0.2 (0.5) |  |
| τ-Methylhistidine | 0.67 ± 0.38 | 1.01 ± 0.58 | 0.8 (0.6) |  |

A significant separation between fasting and postprandial samples was obtained with partial least-squares discriminant analysis (PLS-DA 1 comp: R^2^Y=0.4, Q^2^Y=0.32) and cross-validated analysis of variance (CV-ANOVA: *P*=0.00006) in a multivariate model including all 28 dogs and 45 metabolites (urea and creatinine were excluded).

^a^Relative concentrations were calculated by normalisation of the molar concentration of each metabolite to the total molar concentration of all 45 metabolites (% of total mM).

^b^VIP, Variable importance for the projection; CI, confidence interval; Metabolites with VIP >1 and for which the corresponding jackknife-based 95% CIs were not close to or including zero were considered discriminative and significant for the observed separation.

^c^The Wilcoxon signed-rank test was used for univariate analyses of differences between time points. Only metabolites that were significant from the multivariate model were tested. Level of significance *P*<0.013 after Bonferroni correction.
