# Supplementary material for: The urine metabolome differs between lean and overweight Labrador Retriever dogs during a feed-challenge
Source: PLoS One. 2017 Jun 29;12(6):e0180086. doi: 10.1371/journal.pone.0180086 (PMC5491113; doi:10.1371/journal.pone.0180086)
Supplement: S2 Table — (DOCX) [file pone.0180086.s002.docx]

**Supplementary Table 2. Relative concentrations of urine metabolites in lean and overweight dogs at fasting and postprandial time points**

|  | **Fasting**  **Relative concentration^a^**  *Mean ± SD (% of total mM)* | | **Postprandial**  **Relative concentration^a^**  *Mean ± SD (% of total mM)* | | | |
| --- | --- | --- | --- | --- | --- | --- |
| **Metabolite** | **Lean (n=12)** | **Overweight (n=16)** | **Lean (n=12)** | **Overweight (n=16)** | **VIP (CI)^b^** | ***P*-value^c^** |
| 1-Methylnicotinamide | 1.96 ± 0.91 | 1.57 ± 0.64 | 1.83 ± 0.74 | 1.88 ± 0.59 | 0.05 (0.3) |  |
| 2-Hydroxybutyrate | 0.88 ± 0.23 | 0.99 ± 0.20 | 1.10 ± 0.38 | 1.12 ± 0.31 | 0.04 (0.2) |  |
| 2-Hydroxyphenylacetate | 0.50 ± 0.13 | 0.57 ± 0.13 | 0.69 ± 0.24 | 0.75 ± 0.37 | 0.1 (0.5) |  |
| 2-Methylglutarate | 0.38 ± 0.41 | 0.29 ±0 .10 | 0.52 ± 0.45 | 0.34 ± 0.11 | 0.2 (0.4) |  |
| 2-Oxoglutarate | 2.25 ± 1.02 | 1.98 ± 0.59 | 2.45 ± 0.88 | 2.19 ± 0.80 | 0.4 (0.8) |  |
| 2-Oxoisocaproate | 0.40 ± 0.10 | 0.46 ± 0.06 | 0.50 ± 0.15 | 0.50 ± 0.13 | 0.02 (0.2) |  |
| 2-Oxovalerate | 1.13 ± 0.36 | 1.10 ± 0.26 | 0.94 ± 0.39 | 1.11 ± 0.35 | 0.3 (0.4) |  |
| 3-Hydroxyphenylacetate | 0.41 ± 0.18 | 0.37 ± 0.14 | 0.37 ± 0.16 | 0.38 ± 0.13 | 0.01 (0.2) |  |
| 4-Hydroxyphenylacetate | 0.49 ± 0.28 | 0.50 ± 0.31 | 0.66 ± 0.30 | 0.58 ± 0.39 | 0.2 (0.5) |  |
| Acetamide | 0.61 ± 0.19 | 0.62 ± 0.14 | 0.69 ± 0.23 | 0.70 ± 0.14 | 0.06 (0.3) |  |
| Acetate | 1.38 ± 0.30 | 1.37 ± 0.28 | 1.51 ± 0.87 | 1.42 ± 0.34 | 0.2 (0.6) |  |
| Acetoacetate | 0.50 ± 0.07 | 0.52 ± 0.07 | 0.57 ± 0.12 | 0.63 ± 0.15 | 0.2 (0.2) |  |
| Acetone | 0.35 ± 0.09 | 0.37 ± 0.12 | 0.35 ± 0.08 | 0.39 ± 0.14 | 0.2 (0.1) |  |
| Alanine | 2.01 ± 0.57 | 2.59 ± 0.65 | 1.54 ± 0.34 | 2.07 ± 0.73 | 0.8 (0.7) |  |
| Allantoin | 22.9 ± 6.92 | 24.9 ± 4.66 | 12.8 ± 7.16 | 18.7 ± 5.47 | 2.4 (1.0) | 0.031 |
| Ascorbate | 5.79 ± 3.14 | 6.47 ± 2.47 | 4.31 ± 3.17 | 5.00 ± 2.72 | 0.3 (1.1) |  |
| Betaine | 2.91 ± 2.11 | 3.29 ± 1.31 | 2.95 ± 1.20 | 3.44 ± 1.02 | 0.9 (0.3) |  |
| Choline | 1.03 ± 0.60 | 1.63 ± 1.05 | 1.13 ± 0.58 | 1.70 ± 1.09 | 0.7 (0.7) |  |
| Citrate | 0.93 ± 0.39 | 1.16 ± 0.74 | 2.77 ± 1.92 | 2.46 ± 2.79 | 0.2 (0.5) |  |
| Dimethylamine | 1.53 ± 0.63 | 1.37 ± 0.50 | 1.67 ± 0.43 | 1.64 ± 0.48 | 0.05 (0.4) |  |
| Formate | 0.81 ± 0.35 | 1.13 ± 0.71 | 1.07 ± 0.78 | 1.04 ± 0.62 | 0.01 (0.4) |  |
| Glucose | 2.54 ± 0.91 | 2.57 ± 1.37 | 2.12 ± 0.70 | 2.15 ± 0.70 | 0.01 (0.4) |  |
| Guanidoacetate | 4.96 ± 1.92 | 6.24 ± 2.51 | 5.73 ± 2.25 | 7.49 ± 2.41 | 1.4 (0.9) | 0.11 |
| Hippurate | 1.94 ± 2.42 | 2.19 ± 1.68 | 1.55 ± 1.16 | 2.51 ± 1.81 | 0.9 (1.4) |  |
| Kynurenate | 2.24 ± 1.24 | 3.05 ± 1.63 | 2.85 ± 1.78 | 3.02 ± 1.81 | 0.5 (1.3) |  |
| Lactate | 3.37 ± 1.99 | 2.85 ± 2.18 | 4.67 ± 4.64 | 3.10 ± 2.09 | 1.2 (2.3) |  |
| Malonate | 3.03 ± 1.67 | 3.15 ± 1.68 | 2.63 ± 2.06 | 2.16 ± 1.44 | 0.07 (0.2) |  |
| Methylamine | 0.47 ± 0.25 | 0.55 ± 0.16 | 0.33 ± 0.12 | 0.37 ± 0.10 | 0.1 (0.2) |  |
| Methylguanidine | 0.76 ± 0.20 | 0.72 ± 0.18 | 0.91 ± 0.26 | 0.91 ± 0.22 | 0.1 (0.2) |  |
| Methylmalonate | 3.51 ± 2.92 | 5.75 ± 3.76 | 4.81 ± 4.03 | 7.89 ± 5.28 | 2.2 (2.1) |  |
| Methylsuccinate | 0.53 ± 0.61 | 0.43 ± 0.19 | 0.91 ± 1.11 | 0.45 ± 0.26 | 0.4 (0.6) |  |
| N,N-Dimethylformamide | 0.59 ± 0.16 | 0.63 ± 0.13 | 0.51 ± 0.14 | 0.61 ± 0.11 | 0.4 (0.4) |  |
| N,N-Dimethylglycine | 0.42 ± 0.24 | 0.56 ± 0.33 | 0.28 ± 0.10 | 0.44 ± 0.29 | 0.4 (0.2) |  |
| N-Nitrosodimethylamine | 2.21 ± 0.70 | 2.29 ± 0.70 | 2.69 ± 1.12 | 2.64 ± 0.87 | 0.4 (0.8) |  |
| N-Phenylacetylglycine | 2.14 ± 1.17 | 2.49 ± 1.55 | 2.26 ± 0.93 | 2.85 ± 1.89 | 0.6 (0.9) |  |
| Phenylacetate | 1.22 ± 0.39 | 1.12 ± 0.31 | 0.99 ± 0.34 | 1.26 ± 0.26 | 0.7 (0.5) |  |
| Pyruvate | 1.49 ± 2.99 | 0.65 ± 0.13 | 0.58 ± 0.20 | 0.64 ± 0.15 | 0.2 (0.3) |  |
| Sarcosine | 0.68 ± 0.70 | 0.78 ± 0.64 | 0.50 ± 0.39 | 0.32 ± 0.11 | 0.4 (0.8) |  |
| Succinate | 0.31 ± 0.06 | 0.35 ± 0.16 | 0.37 ± 0.11 | 0.41 ± 0.16 | 0.2 (0.4) |  |
| Tartrate | 0.74 ± 0.26 | 0.65 ± 0.11 | 0.49 ± 0.15 | 0.64 ± 0.18 | 0.5 (0.4) |  |
| Taurine | 15.9 ± 10.6 | 7.67 ± 6.34 | 22.2 ± 8.65 | 9.89 ± 9.07 | 5.0 (1.2) | 0.006 |
| Trigonelline | 0.35 ± 0.21 | 0.35 ± 0.25 | 0.27 ± 0.11 | 0.29 ± 0.14 | 0.08 (0.3) |  |
| Trimethylamine | 0.17 ± 0.04 | 0.17 ± 0.04 | 0.18 ± 0.05 | 0.20 ± 0.05 | 0.1 (0.2) |  |
| Tyrosine | 0.78 ± 0.45 | 0.85 ± 0.34 | 0.72 ± 0.45 | 0.76 ± 0.34 | 0.05 (0.4) |  |
| τ-Methylhistidine | 0.58 ± 0.26 | 0.74 ± 0.45 | 1.05 ± 0.78 | 0.98 ± 0.39 | 0.2 (0.5) |  |

A significant separation between lean and overweight groups at the postprandial time point was obtained with partial least-squares discriminant analysis (PLS-DA 1 comp: R^2^Y=0.5, Q^2^Y=0.36) and cross-validated analysis of variance (CV-ANOVA: *P*=0.005) in a multivariate model including 45 metabolites (urea and creatinine were excluded). No significant separation was observed between lean and overweight dogs at the fasting time point (PLS-DA 1 comp: R^2^Y=0.38, Q^2^Y=0.01 and cross-validated ANOVA: *P*=0.87).

^a^Relative concentrations were calculated by normalisation of the molar concentration of each metabolite to the total molar concentration of all 45 metabolites (% of total mM).

^b^VIP, Variable importance for the projection; CI, confidence interval; Metabolites with VIP >1 and for which the corresponding jackknife-based 95% CIs were not close to or including zero were considered discriminative and significant for the observed separation.

^c^The Mann-Whitney U test was used for univariate analyses of differences between lean and overweight dogs at the postprandial time point. Only metabolites that were significant from the multivariate model were tested. Level of significance *P*<0.017 after Bonferroni correction.
